# DNA methylation as a potential mediator of the association between prenatal tobacco and alcohol exposure and child neurodevelopment in a South African birth cohort

**DOI:** 10.1038/s41398-022-02195-3

**Published:** 2022-09-30

**Authors:** Sarina Abrishamcar, Junyu Chen, Dakotah Feil, Anna Kilanowski, Nastassja Koen, Aneesa Vanker, Catherine J. Wedderburn, Kirsten A. Donald, Heather J. Zar, Dan J. Stein, Anke Hüls

**Affiliations:** 1 Department of Epidemiology, Rollins School of Public Health, Emory University, Atlanta, GA USA; 2 Institute of Epidemiology, Helmholtz Zentrum München - German Research Center for Environmental Health, Neuherberg, Germany; 3 Institute for Medical Information Processing, Biometry, and Epidemiology, Pettenkofer School of Public Health, LMU Munich, Munich Germany; 4 Division of Metabolic and Nutritional Medicine, Dr. von Hauner Children’s Hospital, University of Munich Medical Center, Munich, Germany; 5 Neuroscience Institute, University of Cape Town, Cape Town, South Africa; 6 South African Medical Research Council (SAMRC) Unit on Risk and Resilience in Mental Disorders, University of Cape Town, Cape Town, South Africa; 7 Department of Psychiatry and Mental Health, University of Cape Town, Cape Town, South Africa; 8 Department of Paediatrics and Child Health, Red Cross War Memorial Children’s Hospital, University of Cape Town, Cape Town, South Africa; 9 Department of Clinical Research, London School of Hygiene and Tropical Medicine, London, UK; 10 Gangarosa Department of Environmental Health, Rollins School of Public Health, Emory University, Atlanta, GA USA

**Keywords:** Genomics, Predictive markers

## Abstract

Prenatal tobacco exposure (PTE) and prenatal alcohol exposure (PAE) have been associated with an increased risk of delayed neurodevelopment in children as well as differential newborn DNA methylation (DNAm). However, the biological mechanisms connecting PTE and PAE, DNAm, and neurodevelopment are largely unknown. Here we aim to determine whether differential DNAm mediates the association between PTE and PAE and neurodevelopment at 6 (*N* = 112) and 24 months (*N* = 184) in children from the South African Drakenstein Child Health Study. PTE and PAE were assessed antenatally using urine cotinine measurements and the ASSIST questionnaire, respectively. Cord blood DNAm was measured using the EPIC and 450 K BeadChips. Neurodevelopment (cognitive, language, motor, adaptive behavior, socioemotional) was measured using the Bayley Scales of Infant and Toddler Development, Third Edition. We constructed methylation risk scores (MRS) for PTE and PAE and conducted causal mediation analysis (CMA) with these MRS as mediators. Next, we conducted a high-dimensional mediation analysis to identify individual CpG sites as potential mediators, followed by a CMA to estimate the average causal mediation effects (ACME) and total effect (TE). PTE and PAE were associated with neurodevelopment at 6 but not at 24 months. PTE MRS reached a prediction accuracy (*R*^2^) of 0.23 but did not significantly mediate the association between PTE and neurodevelopment. PAE MRS was not predictive of PAE (*R*^2^ = 0.006). For PTE, 31 CpG sites and eight CpG sites were identified as significant mediators (ACME and TE *P* < 0.05) for the cognitive and motor domains at 6 months, respectively. For PAE, 16 CpG sites and 1 CpG site were significant mediators for the motor and adaptive behavior domains at 6 months, respectively. Several of the associated genes, including *MAD1L1, CAMTA1*, and *ALDH1A2* have been implicated in neurodevelopmental delay, suggesting that differential DNAm may partly explain the biological mechanisms underlying the relationship between PTE and PAE and child neurodevelopment.

## Introduction

Pregnancy is a critical time window for neurodevelopment and a time when the fetus is most susceptible to adverse environmental and prenatal exposures [1]. Prenatal tobacco exposure (PTE) and prenatal alcohol exposure (PAE) during pregnancy are associated with adverse fetal and childhood outcomes. Maternal smoking and alcohol consumption during pregnancy have been associated with adverse fetal outcomes such as low birth weight, sudden infant death syndrome (SIDS), and preterm birth [2–5].

PTE and PAE have also been linked to the etiology of a range of neurodevelopmental disorders in children. For example, PTE has previously been associated with attention deficit hyperactivity disorder (ADHD) [6]. There is evidence that the risk of ADHD increases with higher concentrations of PTE [7]. There is also evidence that maternal smoking during pregnancy is a risk factor for neuropsychiatric disorders such as schizophrenia (SZ) and bipolar disorder (BPD) [8, 9]. PAE is the cause of fetal alcohol spectrum disorder (FASD), a condition characterized by severe neurodevelopmental delay, and which has various manifestations, including growth deficiencies and both behavioral and cognitive deficits [10, 11].

While PTE and PAE have been implicated in the etiology of several neurodevelopmental disorders, the biological mechanisms have yet to be established. Epigenetic modifications, such as differential DNA methylation (DNAm), are dynamic and highly sensitive to external environmental factors [12]. More importantly, DNAm is potentially reversible, indicating that the methylome could be a therapeutic target for disease treatment and prevention [13].

There is strong evidence from large-scale epigenome-wide association studies (EWAS) suggesting that PTE is associated with changes in the cord blood methylome [14–16]. The evidence is weaker for an association between PAE and the cord blood methylome [17], although there has been some evidence of epigenomic changes in other tissues such as placental or buccal tissue [18]. While there is evidence that PTE and PAE influence DNA methylation in cord blood, not much is known about the subsequent adverse outcomes, such as delayed neurodevelopment [19, 20] or about the association between newborn DNAm and child neurodevelopment or related disorders in general [21–23]. Few studies have investigated the mediation of PTE and neuropsychiatric outcomes by DNAm. A candidate gene study found that differential cord blood DNAm levels in *GFI1* acts as a mediator of the association between PTE and ADHD in 6-year-old children [24]. There is also evidence of whole blood DNAm mediating the association between PTE and schizophrenia, as indicated by a meta-analysis of five prospective birth cohorts [25]. While there is evidence of DNAm as a mediator of this relationship with psychiatric outcomes, the epigenetic mechanisms connecting PTE and PAE, cord blood DNAm, and child neurodevelopment are largely unknown.

The majority of EWAS have been conducted in high-income countries (HICs) and/or in children of primarily European ancestry. Despite the rapid proliferation of the field of genomics and translational science in the last decade, cohorts from non-European ancestries and low- and middle-income countries (LMICs) are still largely underrepresented [26]. Consequently, there is a large gap in the literature on these underrepresented populations in which risk factors such as low socioeconomic status, poor maternal health, maternal substance use, and adverse child health outcomes, including neurodevelopmental delay, are at a much higher prevalence [27].

Here, we investigated whether differential DNAm in cord blood mediates the relationship between PTE and PAE and neurodevelopment in children of 6 and 24 months of age using data from a well-characterized South African birth cohort, the Drakenstein Child Health Study (DCHS) [28]. For the causal mediation analysis (CMA), we utilized methylation risk scores (MRS) and high-dimensional mediation analysis (HDMA) to increase statistical power and reduce the dimensionality of the methylation data. To our knowledge, this study is one of the first mediation analyses to examine this relationship in an underrepresented, at-risk, LMIC population, and is also one of the first to utilize HDMA and MRS for epigenetic mediation analysis.

## Materials and methods

### Study population

The DCHS is a South African, population-based birth cohort of African and mixed ancestry and has been previously described [28]. Our analysis sample consisted of 262 children from the DCHS with DNAm data from cord blood, genotype data, PTE and PAE measures, and important covariates. Of these,112 infants had measurements available from the Bayley Scales of Infant and Toddler Development, third edition (BSID-III) at 6 months of age, and 184 had measurements available from BSID-III at 24 months of age. Mothers were enrolled during their second trimester and followed through pregnancy at two primary clinics, Newman and Mbekweni. Mother-child pairs were then followed from birth until the child was at least 5 years of age. Ethical approval for human subjects research was given by the Human Research Ethics Committee of the Faculty of Health Sciences of the University of Cape Town. Written consent for participation was obtained from the mother on behalf of herself and the infant.

### DNA methylation measurements

As described previously, DNA methylation was measured from cord blood using either the Illumina Infinium HumanMethylation450 BeadChip (450 K; *n* = 115) or the Infinium MethylationEPIC BeadChip (EPIC; *n* = 147) arrays [23]. Preprocessing was conducted separately for each array in R 3.5.1, using an identical preprocessing pipeline. The 450 K and EPIC arrays contained 426,378 probes and 781,536 probes, respectively. The 450k and EPIC arrays were combined using the *minfi* package, which resulted in 316 samples and 453,093 probes available in both arrays [29]. Background subtraction, color correction, and normalization were performed using the *preprocessFunnorm* function [30]. After sample and probe filtering, 273 samples and 409,033 probes remained for analysis. Of these samples, there were 262 complete cases with genotype data, PTE and PAE measures, and important covariates. Batch effects were removed using the *ComBat* function from the *sva* package [31]. Cell type composition estimates were calculated using the most recent cord blood reference dataset [32].

### Smoking and alcohol measurements

Prenatal tobacco exposure was objectively measured using urine cotinine levels, which were taken within 4 weeks of enrollment. Urine cotinine was measured using the IMMULITE® 1000 Nicotine Metabolite Kit (Siemens Medical Solutions Diagnostics®, Glyn Rhonwy, United Kingdom) [33]. Urine cotinine levels were classified as non-smoker or passive smoker (<499 ng/ml) or active smoker (≥500 ng/ml). The continuous urine cotinine concentrations were used in this analysis [34].

Prenatal alcohol exposure was measured at the second antenatal study visit using a dichotomous, composite score calculated from the Alcohol, Smoking, and Substance Involvement Screening Test (ASSIST), a self-report questionnaire, and from retrospectively collected data on alcohol consumption during pregnancy [35]. The ASSIST questionnaire was developed by the World Health Organization and has been validated for use in international settings [36, 37]. Details of the composite score calculation have been described elsewhere [35, 38, 39].

### Neurodevelopment measurements

Neurodevelopment was assessed at 6 and 24 months of age using the BSID-III [40, 41]. The BSID-III assessment and its composite scores have been previously validated in South African populations [42, 43]. Trained assessors administered the BSID-III to children via direct observation to generate scores for the cognitive, language, and motor development domains. The same trained assessors administered the BSID-III to mothers to report and generate a score for the adaptive behavior and socioemotional domains [35]. Composite scores generated for each domain were used in this analysis.

### Statistical analysis

First, we estimated the association between PTE and PAE and neurodevelopment at age 6 and 24 months in adjusted linear regression analysis. Mediation analyses with differential DNAm as mediators were conducted using MRS and individual CpG sites as mediators. Significant mediators identified at 6 months of age were validated for neurodevelopment measured at the second timepoint of 24 months.

#### Confounding assessment

Confounding was assessed by constructing directed acyclic graphs (DAGs). DAGs were created for each exposure-mediator (E-M) (PTE/PAE-DNAm), mediator-outcome (M-O) (DNAm-neurodevelopment), and exposure-outcome (E-O) relationship (PTE/PAE-neurodevelopment) (Fig. S1). Potential confounders were selected based on existing literature [35]. All models in this analysis were adjusted for maternal age, maternal HIV status, maternal depression during pregnancy (assessed with the Beck Depression Inventory II (BDI-II)), maternal psychological distress during pregnancy (assessed with the Self Reporting Questionnaire (SRQ-20)), parental socioeconomic status (SES), gestational age, and cell-type proportions. Population stratification was adjusted for using the first five genetic principal components. Models evaluating PTE as the primary exposure were additionally adjusted for PAE and likewise, models evaluating PAE as the primary exposure were additionally adjusted for PTE. Multicollinearity between covariates was evaluated by calculating the variance inflation factors for each predictor using the total effect regression model. There was no evidence of multicollinearity for PTE and PAE (Table S6).

#### Methylation risk scores

First, we constructed methylation risk scores (MRS) for PTE and PAE as described elsewhere [44, 45]. All 262 samples with DNAm data were included in the MRS calculation. MRS are calculated as the weighted sum of methylation beta levels of CpG sites. MRS calculations were based on summary statistics from epigenome-wide association studies (EWAS) for DNAm probes in which the association between the exposure and DNAm was investigated. These summary statistics, which describe the effect size (beta) at each CpG site in the respective EWAS, were used as external weights to calculate the methylation risk score in our cohort. External summary statistics were acquired from EWASs conducted by the Pregnancy and Childhood Epigenetics (PACE) consortium. PTE summary statistics were acquired from an EWAS investigating the association between maternal smoking and cord-blood DNAm (*N* = 5648) [16]. PAE summary statistics were acquired from an EWAS investigating the association between maternal alcohol use and cord-blood DNAm (*N* = 1147) [17]. Both EWASs were comprised of cohorts from primarily European ancestry. To correct for correlated CpG sites, we estimated co-methylated regions (CMR) using the *CoMeBack* package [46]. Then, we conducted “clumping”, in which one CpG site with the smallest *p* value from the external summary statistics per CMR was included in the final MRS. MRS was calculated at several *p* value thresholds for each EWAS, a procedure similar to “thresholding” in traditional polygenic risk score calculations. Finally, we calculated the correlation between the exposure and the resulting MRS at different *p* value thresholds and chose the *p* value threshold associated with the highest prediction accuracy for the subsequent analyses.

#### High-dimensional mediation analyses (HDMA)

MRS are limited in that they only consider the E-M but not the M-O relationship. HDMA considers the E-M as well as the M-O relationship, which allows for a more holistic analysis of the mediating relationships. We performed two separate high-dimensional mediation analyses using the R packages *HIMA* and *DACT* for any exposure-outcome combinations for which we identified indications of a total effect [47, 48]. The *HIMA* package utilizes a penalized-based regression and consists of three steps. First, it performs dimension reduction through sure independence screening to identify the n/log(n) CpG sites with the largest effect size in the mediator-outcome regression model. Second, the minimax concave penalty is applied to this subset of CpG sites for further dimension reduction [49]. Finally, a joint significance test is conducted to evaluate the significance of the mediation effects using a Benjamini–Hochberg false discovery rate (FDR) correction for multiple testing. The divide-aggregate composite null test (DACT) is a more recent method for high-dimensional mediation analysis that is better powered than HIMA [48]. First, it utilizes the Efron empirical null framework to estimate the proportions of the three null cases across all epigenome mediators [50]. Then, it performs the DACT for the composite null of no mediation effect in three cases in which the M-O effect is non-zero, the E-M effect is non-zero, or both effects are non-zero. Finally, the DACT *p* value is calculated as the weighted sum of all three *p* values under the three null hypothesis. We conducted an association analysis for the E-M and M-O associations with robust linear regression using the *rlm* function to input into DACT [51]. We then pre-filtered CpG sites by two criteria to alleviate the burden of multiple testing. First, we filtered the CpG sites by a *p* value threshold of 0.05 for the E-M and M-O models. Second, we filtered CpG sites by the direction of the E-M and M-O effects to achieve an overall negative indirect effect, in line with the hypothesized negative total and direct effects. After running DACT for the resulting subset of CpGs, we corrected for multiple testing using the Benjamini–Hochberg FDR method.

#### Causal mediation analysis (CMA)

Finally, we conducted a CMA for the MRS and significant CpG sites from HDMA as mediators. The *mediation* R package was used to estimate mediation effects [52]. The *mediation* package estimates the average causal mediation effect (ACME), the average direct effect (ADE), the total effect (TE), and the proportion mediated (PM) in the population. The 95% confidence intervals were constructed using a nonparametric, bootstrapped, quasi-Bayesian method and two-sided *p* values were provided for each effect. An α level of 0.05 was used to determine significance [53]. To examine trends across both time points, we cross-validated significant CpG sites identified at 6 months by determining whether they are also significant mediators of the association at 24 months of age.

### Secondary analyses

To further support our findings, we conducted follow-up analyses for any CpG sites that were identified as significant mediators. This included gene ontology (GO) functional enrichment analysis using the *missMethyl* R package [54], blood–brain concordance analysis using the Blood–Brain Epigenetic Concordance (BECon) tool [55], and methylation quantitative trait loci (mQTL) mapping using the GoDMC database [56]. Details are provided in the supplementary methods. Additionally, we compared our findings to previous studies using the publicly available EWAS catalog [57] and conducted further searches on PubMed.

## Results

### Study population characteristics

This analysis sample included 262 children with DNAm data, genotype data, and other relevant covariates, with a subset of 112 children and 184 children with information on neurodevelopment across five domains at 6 months and 24 months, respectively (Table 1). The sample size at each timepoint varied slightly by neurodevelopmental domain and timepoint (Table 1). This is a cohort of children from African (55%) and mixed (45%) ancestry. There was a high prevalence of maternal smoking with 48% of mothers classified as passive smokers and 30% classified as active smokers. The prevalence of prenatal alcohol use among mothers was 17%. Additionally, 25% of mothers were classified as being above the Beck Depression Inventory-II threshold and 30% being at elevated risk of psychological distress on the Self-Reporting Questionnaire (SRQ-20). The prevalence of mothers with an HIV diagnosis was 24%, but all children in this population remained uninfected. Among the subset of children with information on neurodevelopment at 6 months of age, the prevalence of mothers who smoked and reported alcohol use was higher compared to the whole population with DNAm data and children at 24 months, with 35% of mothers classified as active smokers and 22% of mothers classified as consuming alcohol.

**Table 1:** Study population characteristics for the whole sample, children with BSID-III measurements at 6 months of age, and children with BSID-III measurements at 24 months of age.

	Whole study sample	Children with BSID-III data at 6 mo	Children with BSID-III data at 24 mo
***N***	262	112	184
**Maternal age (yrs) (median [IQR])**	26 [22,31]	26 [22,31]	26 [22,32]
**Gestational age (wks) (median [IQR])**	39 [38,40]	39 [38,40]	39 [38,40]
**Child sex (%)**			
Female	116 (44.27)	54 (48.21)	79 (42.93)
Male	146 (55.73)	58 (51.79)	105 (57.07)
**Parental SES**^**a**^ **(%)**			
Lowest SES	66 (25.19)	23 (20.53)	42 (22.83)
Low-moderate SES	64 (24.43)	32 (28.57)	46 (25.00)
Moderate-high SES	73 (27.86)	30 (26.79)	50 (27.17)
Highest SES	59 (22.52)	27 (24.11)	46 (25.00)
**Ancestry (%)**			
Black African	143 (54.58)	52 (46.43)	93 (50.54)
Mixed	119 (45.42)	60 (53.57)	91 (49.46)
**Maternal alcohol use (%)**			
Non-alcohol user	217 (82.82%)	87 (77.68)	150 (81.52)
Alcohol user	45 (17.18%)	25 (22.32)	34 (18.48)
**Maternal smoking**^**b**^ **(%)**			
Non-smoker	59 (22.52)	23 (20.53)	43 (23.37)
Passive Smoker	125 (47.71)	50 (44.64)	79 (42.94)
Active Smoker	78 (29.77)	39 (34.82)	62 (33.70)
**Maternal smoking (ng/ml) (median [IQR])**	38.20 [11.4, 500.00]	57.35 [12.32, 500.00]	54.10 [10.90, 500.00]
**Maternal HIV (%)**			
Yes	64 (24.42)	29 (25.89)	43 (23.00)
No	198 (75.57)	83 (74.11)	144 (77.00)
**Maternal depression during pregnancy**^**c**^ **(%)**			
Above threshold	66 (25.19)	36 (32.14)	48 (26.08)
Below threshold	196 (74.81)	76 (67.86)	136 (73.91)
**Maternal psychological distress during pregnancy**^**d**^ **(%)**			
Above threshold	79 (30.15)	34 (30.36)	57 (30.98)
Below threshold	183 (69.85)	78 (69.64)	127 (69.02)
**Bayley scales 6 months (median [IQR])**			
Cognitive (*n* = 110)	-	105 [95,110]	-
Language (*n* = 109)	-	103 [91,112]	-
Motor (*n* = 110)	-	112 [100,118]	-
Adaptive behavior (*n* = 112)	-	102 [96,107]	-
Social-emotional (*n* = 112)	-	115 [100,125]	-
**Bayley scales 24 months (median [IQR])**			
Cognitive (*n* = 184)	-	-	85 [80,90]
Language (*n* = 173)	-	-	83 [74,91]
Motor (*n* = 173)	-	-	91 [85,100]
Adaptive behavior (*n* = 184)	-	-	84 [71,92]
Social-emotional (*n* = 184)	-	-	115 [100,135]

^a^Socioeconomic status (SES) composite scores (see Supplemental for details).

^b^Urine cotinine levels (ng/ml) classified as non-smoker (<10 ng/ml); passive smoker (10–499 ng/ml); active smoker (≥500 ng/ml).

^c^Assessed with the Beck Depression Inventory II (BDI-II); threshold score of at least 20 indicated moderate depression.

^d^Assessed with the Self-Reporting Questionnaire (SRQ-20); threshold of at least 8 indicated high risk of psychological distress.

### The total effect of prenatal smoking and alcohol exposure on child neurodevelopment

**Fig. 1:**
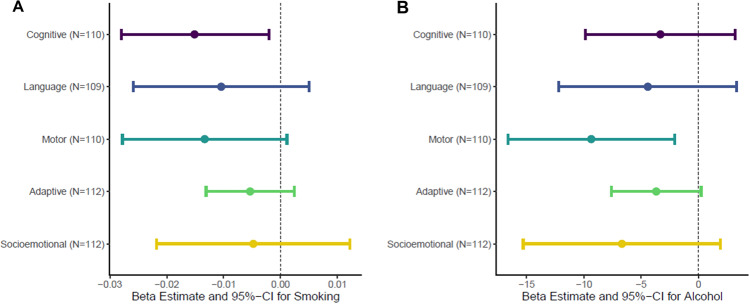
Association of PTE and PAE on neurodevelopment domains at 6 months. Models were adjusted for parental SES, maternal depression, maternal psychological distress, gestational age, maternal age, maternal HIV status, cell-type proportions, and the first five genetic principal components. **A** Association of PTE on neurodevelopment domains, additionally adjusted for PAE. **B** Association of PAE on neurodevelopment domains, additionally adjusted for PTE.

The total effects of PTE and PAE on child neurodevelopment across five domains were estimated using adjusted linear regression (Fig. 1). There was a consistent negative association between PTE and neurodevelopment across all domains at 6 months of age, which was significant for the cognitive domain (β = −0.02; 95% CI: −0.028, 0.002; *P* = 0.023). There was also a consistent negative association between PAE and neurodevelopment across all domains at 6 months of age, which was significant for the motor domain (β = −9.36; 95% CI: −16.64, −2.08; *P* = 0.012). No associations were found between PTE and PAE and neurodevelopment at 24 months in this sample (Fig. S2 and Tables S1, S2). Therefore, the subsequent mediation analyses focused on neurodevelopment at 6 months as the primary outcome. Neurodevelopment at 24 months was used as validation for any significant mediators identified at age 6 months.

### Mediation analyses with methylation risk scores as mediators

**Fig. 2:**
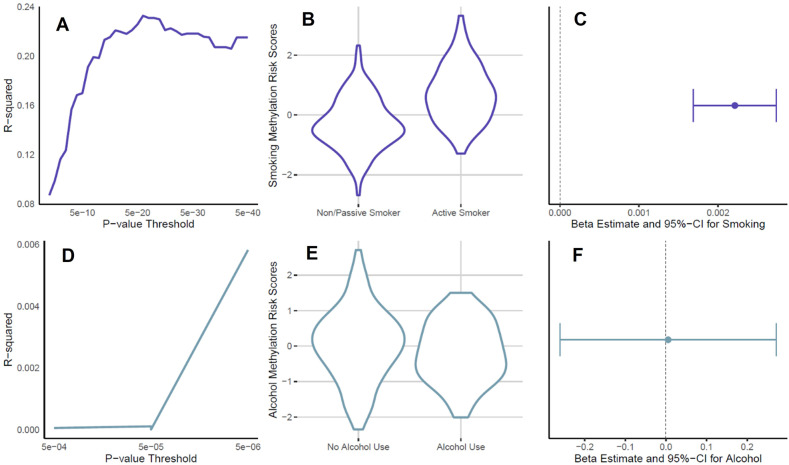
Performance of the MRS for PTE (*N* = 262) and PAE (*N* = 262). **A** Correlation between PTE (continuous maternal urine cotinine levels) and MRS at several *p* value thresholds. **B** MRS (*p* value threshold of 5e-21) for non-/passive smoking mothers and active smoking mothers. **C** Effect of PTE (continuous maternal urine cotinine levels) on most highly correlated MRS (*p* value threshold of 5e-21). **D** Correlation between PAE and MRS at several *p* value thresholds. **E** MRS (*p* value threshold of 5e-06) for non-alcohol consuming mothers and alcohol consuming mothers. **F** Effect of PAE on most highly correlated MRS (*p* value threshold of 5e-06).

The MRS for PTE was well correlated with prenatal cotinine levels, with the highest *R*^2^ being 0.23 at a *p* value threshold of 5e-21 (Fig. 2A). As evident from Fig. 2B, active smokers had a higher MRS than non-smokers and passive smokers combined (β = 0.96, 95% CI: 0.72, 1.21; *P* = 4.01E-13). Additionally, there was a strong and significant association between PTE (continuous maternal urine cotinine levels) and the MRS (β = 0.0022; 95% CI: 0.0016, 0.0027; *P* = 6.7E-15) (Fig. 2C). In contrast, the MRS for PAE was not correlated with alcohol use status, with the highest R [2] being 0.006 at a *p* value threshold of 5e-06 (Fig. 2D). There was no difference between the MRS for children with and without alcohol exposure (Fig. 2E) and there was no association between PAE and the MRS (β = 5.4e-03; 95% CI: −0.26,0.27; *P* = 0.97) (Fig. 2F). Because of the poor performance of the PAE methylation risk score, we did not proceed with CMA for PAE using this approach.

**Fig. 3:**
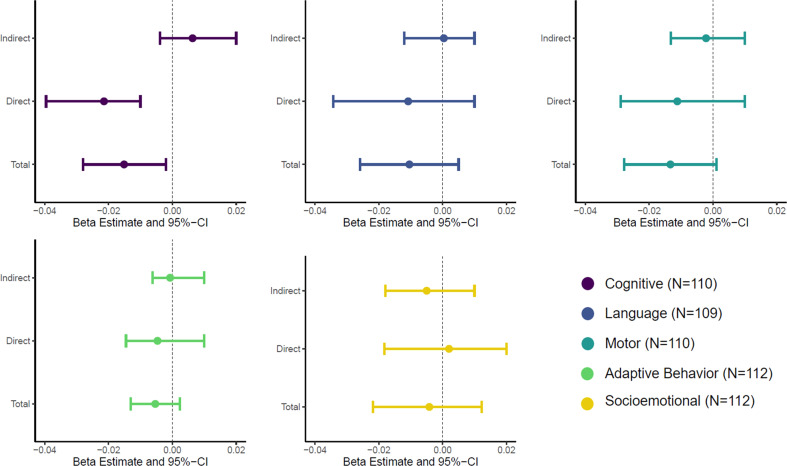
Causal mediation analysis for PTE as the exposure, MRS as the mediator, and neurodevelopment across five domains at 6 months of age as the outcome. Estimated indirect effect, direct effect, and total effect with their corresponding 95% confidence intervals are shown.

There was no evidence of mediation by MRS in any domain (Fig. 3 and Table S3). While the cognitive domain showed a significant total effect, a significant indirect effect (ACME) was not found (ACME = 0.006; 95% CI: −3.84E-03, 0.002; *P* = 0.2). The direct effect was significant, however (ADE = −0.02; 95% CI: −0.04, −0.01; *P* = 0.01).

### HDMA with individual CpG sites as mediators

HDMA was conducted for exposure-outcome combinations for which there was an indication of a total effect: PTE-cognitive, PTE-motor, PAE-motor, and PAE-adaptive behavior. After correcting for multiple testing, DACT identified a total of 191 CpG sites as significant mediators of the association between PTE and neurodevelopment across the cognitive (123 CpG sites) and motor (68 CpG sites) domains. After validating these CpG sites with CMA, we identified 31 CpG sites to have a significant mediating effect (ACME *P* and TE *P* < 0.05) between PTE and cognitive development and eight CpG sites to have a significant mediating effect between PTE and motor development (Table 2). Three of these CpG sites overlapped between both the cognitive and motor domains (cg22263591 [*SLC39A11*], cg25284031 [*HVCN1*], and cg25857569 [*PPM1L*]).

**Table 2:** Significant CpG sites (ACME *P* and TE *P* < 0.05) resulting from HDMA and CMA of DNAm as a potential mediator of the association between PTE and neurodevelopmental delay at 6 months of age using the DACT method and after correction for multiple testing (BH FDR ≤0.05). ACME, ADE, TE, and PM effect sizes and 95% confidence intervals were estimated by CMA.

Domain	CpG (Gene)	ACME (95% CI)	ADE (95% CI)	Total effect (95% CI)	Prop med. (95% CI)
**Cognitive (*n*** = **110)**	cg00480142	−0.004 (−8.9e-03, −5.0e-05)	−0.011 (−0.025, 2.4e-03)	−0.015 (−0.027, −2.4e-03)	0.24 (−0.012, 1.4)
	cg00613752 (*GPR6*)	−0.004 (−9.2e-03, −1.9e-04)	−0.012 (−0.024, 4.2e-04)	−0.016 (−0.029, −3.4e-03)	0.25 (3.2e-03, 0.88)
	cg01258793 (*MAD1L1*)	−0.005 (−0.012, −3.4e-04)	−0.011 (−0.024, 2.3e-03)	−0.016 (−0.030, −2.7e-03)	0.31 (5.5e-03, 1.3)
	cg02215141	−0.004 (−9.6e-03, −5.5e-05)	−0.011 (−0.024, 2.5e-03)	−0.016 (−0.028, −2.5e-03)	0.28 (−0.022, 1.3)
	cg02423105 (*CDH26*)	−0.005 (−0.012, −6.0e-05)	−0.014 (−0.026, −1.2e-03)	−0.018 (−0.031, −4.8e-03)	0.26 (1.1e-03, 0.83)
	cg02668773 (*MPO*)	−0.007 (−0.014, −7.7e-04)	−0.012 (−0.025, 1.4e-03)	−0.019 (−0.033, −4.8e-03)	0.36 (0.05, 1.1)
	cg02879122(*CCDC144A*)	−0.004 (−9.3e-03, −3.8e-04)	−0.012 (−0.025, 1.2e-03)	−0.016 (−0.028, −2.9e-03)	0.26 (0.018, 1.2)
	cg03288340 (*ZC3H4*)	−0.005 (−0.012, −6.3e-04)	−0.011 (−0.023, 2.1e-03)	−0.016 (−0.029, −3.2e-03)	0.33 (0.039, 1.3)
	cg05129050 (*CAMTA1*)	−0.005 (−0.011, −6.9e-04)	−0.010 (−0.023, 4.5e-03)	−0.014 (−0.027, −1.4e-03)	0.35 (9.1e-03, 2.0)
	cg05331340 (*ASGR1*)	−0.010 (−0.020, −1.8e-03)	−0.012 (−0.025, 7.9e-04)	−0.022 (−0.037, −7.6e-03)	0.44 (0.093, 1.0)
	cg08481075 (*RXRB, COL11* *A2*)	−0.005 (−0.011, −2.9e-04)	−0.012 (−0.024, 1.9e-03)	−0.016 (−0.029, −2.9e-03)	0.28 (2.5e-03, 1.1)
	cg08836481 (*LARP4B*)	−0.008 (−0.016, −1.2e-03)	−0.012 (−0.025, 4.2e-04)	−0.020 (−0.034, −6.2e-03)	0.39 (0.08, 1.0)
	cg09115275 (*IL2RA*)	−0.004 (−8.3e-03, −2.2e-04)	−0.012 (−0.024, 1.5e-03)	−0.015 (−0.027, −2.3e-03)	0.23 (−0.015, 1.1)
	cg09964326 (*FGFBP3*)	−0.004 (−0.010, −4.5e-04)	−0.012 (−0.025, 1.2e-03)	−0.017 (−0.030, −3.3e-03)	0.27 (0.024, 1.2)
	cg10149329 (*C4orf29*, *MFSD8*)	−0.004 (−9.2e-03, −3.7e-04)	−0.011 (−0.024, 3.0e-03)	−0.015 (−0.027, −1.9e-03)	0.27 (−2.7e-03, 1.4)
	cg10780097 (*AMD1*)	−0.003 (−8.0e-03, −1.6e-05)	−0.012 (−0.024, −5.9e-04)	−0.015 (−0.028, −2.6e-03)	0.20 (−5.9e-03, 0.76)
	cg12031962 (*ALDH1A2*)	−0.004 (−0.010, −3.5e-04)	−0.011 (−0.024, 3.1e-03)	−0.016 (−0.029, −2.5e-03)	0.26 (0.015, 1.4)
	cg12428160	−0.005 (−0.014, −4.3e-04)	−0.013 (−0.025, 7.9e-04)	−0.018 (−0.031, −4.0e-03)	0.28 (0.025, 1.1)
	cg13889963 (*CALB1*)	−0.004 (−9.4e-03, −8.2e-05)	−0.011 (−0.024, 2.1e-03)	−0.015 (−0.028, −2.2e-03)	0.28 (−0.039, 1.2)
	cg13918314 (*ANAPC13*)	−0.004 (−9.8e-03, −2.1e-05)	−0.013 (−0.025, −6.1e-04)	−0.017 (−0.030, −3.4e-03)	0.22 (−5.5e-03, 0.84)
	cg14323109 (*KDR*)	−0.004 (−9.1e-03, −8.4e-05)	−0.011 (−0.023, 3.1e-03)	−0.015 (−0.027, −9.5e-04)	0.24 (−0.01, 1.6)
	cg14896134	−0.007 (−0.016, −6.0e-04)	−0.010 (−0.023, 2.0e-03)	−0.017 (−0.030, −3.8e-03)	0.40 (0.035, 1.2)
	cg15624376 (*HOXA4*)	−0.004 (−0.010, −2.2e-04)	−0.012 (−0.025, 7.9e-04)	−0.016 (−0.029, −2.6e-03)	0.25 (1.7e-03, 1.1)
	cg18780259 (*CERS1, GDF1*)	−0.005 (−0.012, −5.1e-04)	−0.010 (−0.022, 2.8e-03)	−0.015 (−0.028, −2.3e-03)	0.34 (0.022, 1.3)
	cg19611886	−0.009 (−0.021, −8.2e-04)	−0.012 (−0.025, 1.6e-03)	−0.021 (−0.035, −6.2e-03)	0.42 (0.034, 1.1)
	cg22263591 (*SLC39A11*)	−0.006 (−0.014, −3.9e-04)	−0.013 (−0.025, −4.9e-04)	−0.019 (−0.031, −5.1e-03)	0.33 (0.022, 0.89)
	cg23219570 (*FGF23*)	−0.007 (−0.016, −4.5e-04)	−0.011 (−0.024, 1.5e-03)	−0.019 (−0.032, −5.6e-03)	0.40 (0.021, 1.1)
	cg23643526 (*HAO2*)	−0.004 (−9.5e-03, −1.3e-04)	−0.011 (−0.024, 3.0e-03)	−0.015 (−0.027, −2.2e-03)	0.25 (−0.022, 1.4)
	cg25284031 (*HVCN1*)	−0.006 (−0.015, −2.2e-04)	−0.014 (−0.026, −5.0e-04)	−0.019 (−0.033, −5.8e-03)	0.30 (9.9e-03, 0.91)
	cg25823085 (*ABCB4*)	−0.004 (−9.1e-03, −2.0e-04)	−0.011 (−0.023, 2.2e-03)	−0.014 (−0.027, −1.5e-03)	0.27 (−0.021, 1.2)
	cg25857569 (*PPM1L*)	−0.007 (−0.015, −1.6e-04)	−0.012 (−0.025, 5.7e-05)	−0.019 (−0.033, −5.1e-03)	0.36 (5.5e-03, 0.94)
**Motor (*n*** = **110)**	cg00328376 (*SKAP2*)	−0.010 (−0.03, −4.9e-03)	−0.010 (−0.023, 9.0e-03)	−0.023 (−0.042, −4.6e-03)	0.65 (0.26, 2.1)
	cg09490277 (*FANCE*)	−0.010 (−0.024, −5.3e-04)	−0.011 (−0.026, 4.4e-03)	−0.022 (−0.043, −3.1e-03)	0.49 (0.019, 1.4)
	cg14989316 (*ZMIZ1-AS1*)	−0.008 (−0.017, −1.6e-03)	−0.010 (−0.025, 6.5e-03)	−0.018 (−0.035, −1.9e-04)	0.46 (−7.2e-03, 2.9)
	cg15188623 (*ZNF710*)	−0.008 (−0.021, −6.8e-04)	−0.010 (−0.025, 6.0e-03)	−0.018 (−0.037, −8.3e-04)	0.46 (−0.015, 2.1)
	cg22263591 (*SLC39A11*)	−0.006 (−0.014, −2.8e-05)	−0.011 (−0.026, 4.8e-03)	−0.017 (−0.032, −3.6e-04)	0.35 (−0.074, 2.0)
	cg24671734 (*BTBD11*)	−0.007 (−0.019, −6.7e-04)	−0.011 (−0.027, 6.1e-03)	−0.018 (−0.034, −1.9e-03)	0.41 (−0.014, 2.0)
	cg25284031 (*HVCN1*)	−0.007 (−0.017, −3.1e-04)	−0.012 (−0.026, 4.6e-03)	−0.018 (−0.035, −1.9e-03)	0.36 (−0.037, 1.8)
	cg25857569 (*PPM1L*)	−0.010 (−0.023, −2.9e-03)	−0.010 (−0.024, 6.8e-03)	−0.020 (−0.037, −2.6e-03)	0.57 (0.17, 1.9)

DACT identified 193 CpG sites as significant mediators of the association between PAE and neurodevelopment across the motor (100 CpG sites) and adaptive behavior (93 CpG sites) domains. After conducting CMA, this resulted in 16 CpG sites and 1 CpG site with a significant mediating effect between PAE and the motor and adaptive behavior domains, respectively (Table 3).

**Table 3:** Significant CpG sites (ACME *P* and TE *P* < 0.05) resulting from HDMA and CMA of DNAm as a potential mediator of the association between PAE and neurodevelopmental delay at 6 months of age using the DACT method and after correction for multiple testing (BH FDR ≤0.05). ACME, ADE, TE, PM and corresponding 95% confidence intervals estimated by CMA are shown.

Domain	CpG (Gene)	ACME (95% CI)	ADE (95% CI)	Total effect (95% CI)	Prop med. (95% CI)
**Motor (*n*** = **110)**	cg00086871 (*TBC1D14*)	−1.8 (−4.4, −0.07)	−8.1 (−15.4, −0.83)	−9.9 (−17.1, −2.9)	0.18 (4.6e-03, 0.69)
	cg00512279 (*SLC18A2*)	−2.5 (−6.1, −0.21)	−7.3 (−14.5, 0.46)	−9.8 (−16.7, −2.9)	0.25 (0.02, 1.1)
	cg02230180 (*HNRNPU*)	−2.6 (−6.1, −0.14)	−7.4 (−14.8, 0.49)	−10.0 (−16.8, −2.9)	0.26 (0.02, 1.1)
	cg03113572 (*ZNF331*)	−3.5 (−7.4, −0.59)	−5.9 (−12.7, 1.0)	−9.3 (−16.5, −2.2)	0.37 (0.06, 1.3)
	cg04743947	−2.7 (−6.7, −0.03)	−6.7 (−14.3, 0.85)	−9.4 (−16.5, −2.6)	0.29 (2.8e-03, 1.2)
	cg04875987 (*LRRC45, STRA13*)	−3.0 (−6.9, −0.29)	−6.3 (−13.5, 1.1)	−9.2 (−16.2, −2.2)	0.32 (0.03, 1.2)
	cg05141711 (*AGMAT*)	−2.8 (−6.7, −0.28)	−6.9 (−13.7, −0.11)	−9.7 (−16.5, −2.9)	0.29 (0.03, 0.9)
	cg09528884 (*KLLN, PTEN*)	−4.0 (−8.2, −1.30)	−6.7 (−13.5, 5.3e-03)	−10.6 (−17.7, −3.8)	0.37 (0.13, 0.97)
	cg10137084 (*CACNA2D3*)	−3.3 (−7.9, −0.13)	−6.6 (−13.2, 0.99)	−9.8 (−16.5, −2.8)	0.33 (5.6e-03, 1.3)
	cg12653570	−2.7 (−6.5, −0.19)	−7.1 (−13.5, −0.49)	−9.8 (−16.9, −2.9)	0.27 (0.02, 0.78)
	cg12789343	−2.0 (−4.8, −0.04)	−8.0 (−15.1, −1.2)	−10.0 (−16.8, −3.1)	0.20 (5.9e-03, 0.64)
	cg19340420 (*RCN3*)	−2.9 (−6.1, −0.49)	−7.4 (−14.8, −0.64)	−10.3 (−17.1, −3.3)	0.28 (0.05, 0.86)
	cg22804663 (*TDG*)	−3.0 (−6.4, −0.09)	−6.6 (−13.9, −0.04)	−9.7 (−16.9, −2.5)	0.31 (−3.1e-03, 0.96)
	cg23238147 (*KAZN, TMEM51-AS1*)	−2.3 (−5.4, −0.11)	−6.8 (−14.0, 0.23)	−9.2 (−16.1, −2.3)	0.25 (3.0e-03, 1.0)
	cg24716343 (*PCNX2*)	−2.8 (−6.2, −0.30)	−6.2 (−12.8, 1.20)	−9.0 (−16.0, −1.9)	0.31 (0.04, 1.3)
	cg26848718 (*WT1*)	−3.4 (−8.3, −0.23)	−5.4 (−11.9, 1.30)	−8.8 (−15.7, −2.0)	0.39 (0.02, 1.3)
**Adaptive behavior (*n*** = **112)**	cg21553656 (*DAG1*)	−2.1 (−4.4, −0.35)	−2.3 (−5.98, 1.90)	−4.3 (−8.2, −0.3)	0.48 (0.03, 2.0)

The second HDMA method we tested, HIMA, did not identify any CpG sites as potential mediators of the association between either PTE and PAE and neurodevelopment at 6 months of age after correction for multiple testing.

None of the CpG sites identified as significant mediators for neurodevelopment at 6 months mediated the association between PTE or PAE and neurodevelopment at 24 months (Tables S4, S5).

### Secondary analyses

After correction for multiple testing (FDR <0.05), we did not identify any GO terms or KEGG pathways with an overrepresentation of genes containing significantly, differentially methylated CpGs that would indicate an enriched biological pathway. The top GO terms and KEGG pathways for each E-O model are included in the supplement (Table S7a, b).

Of the 31 CpG sites identified as significant mediators of the association between PTE and cognitive development, 19 were associated with at least one mQTL (Table S8). Seven CpG sites exhibited blood–brain concordance in accordance with the criteria listed in the methods (Table S9). CpG sites associated with the genes *ALDH1A2* (75–90%), *GPR6* (75–90%), and *HVCN1* (90%) exhibited the highest percentile mean correlations.

Of the eight CpG sites identified as significant mediators of the association between PTE and motor development, six were associated with at least one mQTL (Table S8). Here, six CpG sites exhibited blood–brain concordance, with the highest percentile mean correlations seen at CpG sites associated with the genes *FANCE* (75–90%), *HVCN1* (90%), *ZMIZ1-AS1* (75–90%), and *ZNF710* (90%) (Table S9).

For the 16 CpG sites identified as significant mediators of the association between PAE and motor development, six were associated with an mQTL. No CpG sites exhibited blood–brain concordance for the association between PAE and motor development (Table S9).

There were no mQTLs associated with the CpG site identified as a significant mediator of the association between PAE and adaptive behavior development (Table S8). Additionally, the probe did not exhibit blood–brain concordance (Table S9).

## Discussion

In this South African cohort, we found a significant negative association (total effect) between PTE and PAE and neurodevelopment of exposed children at 6 months of age, as well as evidence of epigenetic mediation of this association using high-dimensional mediation analysis (DACT). We identified 39 CpGs that mediated the association between PTE and child cognitive (31 CpGs) and motor (eight CpGs) development. We also identified 16 CpG sites and one CpG site that mediated the association between PAE and child motor and adaptive behavior development, respectively. While the PTE MRS was highly predictive of PTE, it did not mediate the association between PTE and neurodevelopment. We did not find any evidence of epigenetic mediation of PTE and PAE and neurodevelopment in children of 24 months of age, most likely due to the absence of a total effect of PTE and PAE on neurodevelopment at 24 months in this sample.

Several studies have investigated the association between PTE and PAE and neurodevelopment and related disorders [58–60]. One study (*N* = 446) conducted in a rural region of China in children with an average age of 15 months found that prenatal exposure to tobacco smoke was associated with neurodevelopmental delay in the cognitive and language domains, assessed using the BSID-III [61]. While we did find evidence of an association between PTE and cognitive development, associations with language development were not statistically significant, although it may be difficult to detect language differences as early as 6 months [43, 62]. Prenatal alcohol exposure has been previously associated with decreased gross motor function, but findings have been inconsistent [63, 64]. For example, a study (*N* = 1324) that utilized the BSID-III to evaluate the relationship between PAE and gross motor development in infants of 12 months did not find evidence of an association [65]. However, the prevalence of moderate to severe PAE was lower in this cohort than in the DCHS. In our study, we found associations between PTE and PAE and neurodevelopment at age 6 months but not at age 24 months. This is in line with findings from a previous study conducted in a larger subset of the DCHS (*N* = 734), in which PAE was associated with delayed motor development at 6 months, but not at 24 months [66]. Previous literature on neurodevelopment at 6 months of age is sparse, particularly in association with prenatal exposures. Most studies had an average infant age of at least 12 months. Unlike our present analysis, previous studies have found associations between PTE and PAE and neurodevelopment at or around 24 months of age or older [59, 60, 67].

For example, a study conducted in a Polish birth cohort (*N* = 461) identified a statistically significant association between prenatal tobacco smoke exposure and delayed development in the cognitive, language, and motor domains at 24 months of age using the BSID-III [68]. A potential explanation for this discrepancy is our relatively small sample size (*N* = 112 at age 6 months and *N* = 184 at age 24 months). Several of the CpG sites and associated genes that we identified through HDMA are well known for their association with sustained maternal smoking during pregnancy across two major studies from the PACE consortium [69]. Differential methylation at one of the CpG sites (cg23219570) and four genes (*MAD1L1*, *CAMTA1*, *LARP4B*, *FGF23*) that were identified as mediators for associations with cognitive development was previously identified in one PACE study which investigated the association of sustained prenatal smoking on changes in cord blood DNAm (450 K) across nine cohorts (*N* = 5648) [16]. Differential DNAm at two CpG sites (cg02668773 and cg12031962) and seven genes (*MAD1L1, MPO, CAMTA1, COL11A2, LARP4B, ALDH1A2,* and *SLC39A11*) in association with cognitive development and one CpG site (cg24671734) and four genes (*SKAP2, ZNF710, SLC39A11,* and *BTBD11*) in association with motor development which we identified as mediators were previously identified in a more recent PACE study, which investigated the effects of sustained prenatal smoking on cord blood DNAm (450 K) across 13 cohorts (*N* = 6685) [14]. It is notable that the genes *MAD1L1*, *CAMTA1*, and *LARP4B* had significantly, differentially methylated CpG sites across both PACE studies and our study.

Four of these genes have further been shown to be associated with cognitive outcomes. Aldehyde dehydrogenase 1A2 (*ALDH1A2*) is associated with autism spectrum disorder (ASD) in children [70]. The *ALDH* family of genes plays a key role in the metabolism of vitamin A, an essential molecule for neuronal differentiation and development [71]. Additionally, the identified probe (cg12031962) had high blood–brain concordance. However, there has been inconsistent evidence of an association between maternal smoking and ASD [72–74]. Furthermore, brain tissue-based DNAm in two of these genes (*MAD1L1* and *COL11A2)* was associated with ASD, indicating their role in neurodevelopment [75].

Mitotic arrest deficient 1 like 1 (*MAD1L1)* is a key regulatory gene of the cell cycle. It has been implicated in the etiology of neuropsychiatric disorders such as SZ and BPD in several genome-wide association studies (GWAS) and candidate gene studies, including one study which found altered levels of expression of *MAD1L1* in human neural progenitor cells [76–78]. The biological mechanisms of both SZ and BPD are linked to changes in the mesolimbic reward pathway and high levels of MAD1L1 expression have been found in brain regions such as the ventral tegmental area [79]. However, the identified probe (cg01258793) did not exhibit blood–brain concordance using the BECon tool. Previous studies have found that maternal smoking was associated with a higher rate of SZ and BPD in offspring, suggesting that differential expression of MAD1L1 may be a plausible pathway that links smoking to neurodevelopmental disorders [80].

Calmodulin-binding transcription activator 1 (*CAMTA1*) is also involved in cell cycle regulation and is highly expressed in the brain [81]. Studies have found that *CAMTA1* plays a role in memory production and recall, dysfunction of which is implicated in disorders such as down syndrome, schizophrenia, and depression [82, 83]. However, these studies have been conducted in older adults, and more research needs to be done to corroborate memory dysfunction in children with differential expression of *CAMTA1*.

Differential methylation at two CpG sites that we identified as significant mediators of the association between PAE and motor development mapped to *SLC18A2* (cg00512279) and *HNRNPU* (cg02230180) [84]. Homozygous mutations in *SLC18A2*, a vesicular monoamine transporter, have been implicated in the presentation of infantile movement disorder also known as infantile hypotonic parkinsonian disorder or brain dopamine-serotonin vesicular transport disease [85–87]. Mutations in *HNRNPU* have been associated with infantile spasms [88, 89]. However, there has been little to no research published on prenatal and early life exposures or pediatric movement disorders and related epigenetic modifications.

Methylation risk scores can be used as a potential biomarker for prenatal smoking and alcohol use and to reduce the dimensionality of methylation data, giving us more statistical power to detect associations [44]. Our MRS for prenatal smoking was highly predictive of PTE and is comparable to a previously established MRS for PTE by Reese et al. (*R*^2^ = 0.23) and outperforms another established MRS for PTE by Richmond et al. (*R*^2^ = 0.14) [45, 90, 91]. However, our MRS for PTE did not significantly mediate the association between PTE and neurodevelopment. One possible explanation is that our MRS algorithm only considers the relationship between exposure and mediator, whereas HDMA considers both the E-M and M-O relationships. Additionally, it is possible that the difference in ancestry between the external studies (primarily European ancestry) and the DCHS (African and mixed ancestry) reduced the prediction accuracy of our MRS for both prenatal smoking and prenatal alcohol use [92].

Our study has a few limitations. First, unlike PTE, which has been extensively studied [14–16, 93] in association with DNAm, the association between PAE and DNAm is not well understood, which makes the comparison of our findings to existing literature more challenging. The vast majority of epigenetic studies evaluating the effects of prenatal alcohol exposure are conducted in birth cohorts from HICs and tend to have a low prevalence of PAE [17, 69]. Because of the low prevalence of the exposure, these studies often do not have the statistical power to detect an association. A PACE study that investigated the association between PAE and cord blood DNAm (450 K) across six cohorts (*N* = 1147) did not find strong evidence of a relationship [17]. This limitation also in part explains the poor performance of our MRS for PAE. Furthermore, although the DCHS has a higher prevalence of both PAE and PTE compared to HIC cohorts, this study had a relatively small sample size at both 6 months (*N* = 112) and 24 months (*N* = 184), which may have limited our statistical power to detect associations between PTE and PAE and neurodevelopment across other domains and at 24 months of age.

Another limitation of our study is the possible presence of exposure misclassification bias. Underreporting is a common problem when attempting to ascertain socially undesirable behaviors such as smoking or alcohol consumption during pregnancy. In our case, alcohol use was measured using a self-report questionnaire and smoking was measured via urine cotinine levels. Although using cotinine as a biomarker of smoking is not affected by social desirability bias, it was only measured at one timepoint in this cohort. Exposure misclassification can be an issue in epigenomic mediation analyses in that the DNA methylation estimates may be a more accurate indicator of exposure than the variables available in our cohort, which can lead to an overestimation of the mediation effect, an underestimation of the direct effect, and an increased type I error rate [94]. Because of this potential for bias, our results should be interpreted with caution and methods to correct for misclassification should be considered in future studies.

Furthermore, while blood is the most common tissue used to evaluate the epigenomic mechanisms of a multitude of risk factors and outcomes, it is not yet clear whether blood tissue can be effectively used to evaluate neurological outcomes. Epigenetic signatures are tissue and cell-type specific, and as such, the selection of relevant tissues and cell-type is crucial for epigenetic studies. While brain tissue would be the most relevant tissue to study in association with neurodevelopment, cord blood has the advantage in that it is more easily accessible from living individuals and could therefore be linked to future cognitive outcomes [95]. Furthermore, many of the CpG sites we identified as significant mediators of the association between PTE and neurodevelopment showed good blood–brain concordance (assessed using the BECon tool [55]).

Lastly, many of our significant CpG sites were associated with at least one known mQTL, which is an indicator of the genetic influence on DNAm levels [56]. While we cannot decompose the effects of genetic and environmental factors on DNAm levels, only a proportion of the variation in DNAm levels is explained by genetic effects. In fact, the joint effects of single nucleotide polymorphisms (SNP) and environmental factors have been found to be larger contributors to DNAm variation than SNPs alone [96, 97]. Since the environmental exposures, PTE and PAE, were ascertained before cord blood was collected, and neurodevelopment was ascertained at age 6 and 24 months, reverse causation is unlikely to be a problem in this study. Our study has several strengths. To start, we have data at 6 months of age, which may allow us to infer a causal relationship more confidently because of the short temporality between birth and measurement. Further, while this analysis had a small sample size, the use of novel methods to increase our statistical power allowed us to detect more potentially meaningful associations and is an important strength. To our knowledge, this is one of the first epigenomic studies to employ HDMA. The HIMA method, which utilizes the joint significance test, was potentially too conservative and underpowered to detect significant mediators in this study [48]. DACT has been shown to be more powered than HIMA. The use of methylation risk scores and high-dimensional mediation analysis in the field is still in its infancy. We recognize that more studies will be required to validate the robustness of these methods as the field advances, but this study provides some insight into the utility and potential of HDMA in real-life settings.

Finally, our analysis was conducted on a subset of a well-characterized, underrepresented, birth cohort from an LMIC. We were able to detect associations due to the high prevalence of behavioral risk factors—such as maternal smoking and alcohol consumption- in such vulnerable populations, a characteristic that cohorts from HICs typically do not exhibit. This highlights the importance of conducting large-scale multi-ethnic cohort studies to advance the field of (epi)genomics and reduce health disparities. As Wojcik et al. point out, while the burden of disease disproportionality lies with marginalized populations, the majority of studies are conducted in populations of European ancestry, which limits the field’s ability to investigate the burden of disease in vulnerable communities [98]. As follows, investigating epigenomic associations with health outcomes in a well-characterized cohort from an LMIC allows us to add to the sparse literature and shed light on the importance of prioritizing cohorts like the DCHS.

Overall, these findings suggest that epigenetic changes in the methylome may in part explain the biological mechanisms underlying the relationship between maternal smoking and alcohol consumption during pregnancy and child neurodevelopment across the cognitive, motor, and adaptive behavior domains. Our study provides motivation to conduct larger mediation studies to replicate our findings. Mediation analyses in epigenomics are important for discerning causal mechanisms from exposure to disease. The identification of significant CpG sites could provide novel insights for the early detection of disease and potential prevention targets in translational research and community interventions in at-risk populations.

## Supplementary information

Supplementary Methods and Figures

Supplementary Tables

## Data Availability

All software and packages used for statistical analyses are freely available through the following links: R (V.4.0.3 https://www.r-project.org/); CoMeBack package (V.1.0.1 https://bitbucket.org/flopflip/comeback/src/master/); MRS pipeline (V.0.1.0 https://github.com/jche453/Pruning-Thresholding-MRS); HIMA package (V.2.0.1 https://cran.r-project.org/web/packages/HIMA/index.html); DACT package (V.0.1.0 https://github.com/zhonghualiu/DACT/); mediation package (V.4.5.0 https://cran.r-project.org/web/packages/mediation/vignettes/mediation.pdf).
