# Supplementary material for: DNA methylation as a potential mediator of the association between prenatal tobacco and alcohol exposure and child neurodevelopment in a South African birth cohort
Source: Transl Psychiatry. 2022 Sep 30;12:418. doi: 10.1038/s41398-022-02195-3 (PMC9525659; doi:10.1038/s41398-022-02195-3)
Supplement: Supplementary file 1 — Supplementary Methods and Figures [file 41398_2022_2195_MOESM1_ESM.docx]

# Supplementary Material

DNA Methylation as a Potential Mediator of the Association Between Prenatal Tobacco and Alcohol Exposure and Child Neurodevelopment in a South African Birth Cohort

## Sarina Abrishamcar^1^, Junyu Chen^1^, Dakotah Feil^1^, Anna Kilanowski^2,3,4^, Nastassja Koen^5,6,7^, Aneesa Vanker^8^, Catherine J. Wedderburn^5,8,9^, Kirsten A. Donald^5,8^, Heather J. Zar^6,8^, Dan J. Stein^5,6,7^, Anke Hüls^1,10^

^1^Department of Epidemiology, Rollins School of Public Health, Emory University, Atlanta, Georgia, USA​

^2^Institute of Epidemiology, Helmholtz Zentrum München - German Research Center for Environmental Health, Neuherberg, Germany

^3^Institute for Medical Information Processing, Biometry, and Epidemiology; Pettenkofer School of Public Health, LMU Munich, Munich, Germany

^4^Division of Metabolic and Nutritional Medicine, Dr. von Hauner Children's Hospital, University of Munich Medical Center, Munich, Germany

^5^Neuroscience Institute, University of Cape Town, Cape Town, South Africa​

^6^South African Medical Research Council (SAMRC) Unit on Risk and Resilience in Mental Disorders, University of Cape Town, Cape Town, South Africa​

^7^Department of Psychiatry and Mental Health, University of Cape Town, Cape Town, South Africa​

^8^Department of Paediatrics and Child Health, Red Cross War Memorial Children’s Hospital, University of Cape Town, South Africa​

^9^Department of Clinical Research, London School of Hygiene and Tropical Medicine, London, UK

^10^Gangarosa Department of Environmental Health, Rollins School of Public Health, Emory University, Atlanta, Georgia, USA​

# Supplementary Methods

## **DNA Methylation Quality Control and Processing**

DNA was isolated from cord blood samples that were collected at time of delivery ^1^. DNA methylation was assessed with the Illumina Infinium HumanMethylation450 BeadChips (n=156) and the MethylationEPIC BeadChips (n=160). Pre-processing and statistics were done using R 3.5.1 ^2^. Raw iDat files were imported to RStudio where intensity values were converted into beta values. The 450K and EPIC datasets were then combined using the minfi package ^3^ resulting in 316 samples and 453,093 probes. Background subtraction, color correction and normalization were performed using the preprocessFunnorm function ^4^. After sample and probe filtering, 273 samples and 409,033 probes remained for downstream analyses. Batch effects were removed using ComBat from the R package sva ^5^. Cord blood cell type composition was predicted using the most recent cord blood reference data set ^6^ and the IDOL algorithm and probe selection ^7^.

Sample Filtering

Samples were determined to be outliers if detected using two or more of the following methods: detectOutlier function from the lumi package ^8^, Hannum et al. (2013) method ^9^ using the locFDR package ^10^ and both the outlyx and pfilter functions from the watermelon package ^11^. However, no samples we detected in more than one method and so none were removed for this reason. Samples containing maternal blood contamination (n = 33) were removed ^1^. After the completion of pre-processing technical replicates (n = 7) and samples where reported sex didn’t match sex chromosome methylation signatures (n = 3) were removed leaving a total of 273 samples remaining for downstream analysis.

Probe Filtering

This dataset contains 59 probes which detect single nucleotide polymorphisms for quality control purposes and so once observed, were removed. Probes with NAs in ≥ 1% of samples or had a detection p value ≥ 1x10^-16^ in ≥ 1% of samples were removed (n = 10,868). Probes which bind to the sex chromosomes were removed due to the distribution differences observed (n = 9,896). Probes whose sequence contains a SNP either at the CpG site being measured or at the site of the single base pair extension with a minor allele frequency ≥ 1% ^12,13^ were removed (n = 13,598). Autosomal probes which were in silico predicted to non-specifically bind to sex chromosomes in the genome were also removed (n = 9,698) leaving a total of 409,033 probes remaining for downstream analysis ^12,13^

## **Secondary Analyses**

#### Gene Ontology Enrichment Analysis

To identify potential biological pathways that may be enriched by differential DNAm, we conducted gene ontology functional enrichment analysis using the gometh function of the *missMethyl* R package^14^. We utilized both the Gene Ontology (GO) and Kyoto Encyclopedia of Genes and Genomes (KEGG) gene set collections available from the R package. *Gometh* identifies GO terms and KEGG pathways that are overrepresented among genes containing differentially methylated CpG sites.

Blood-Brain ConcordanceBlood-brain concordance for significant CpG sites was assessed using the publicly available Blood-Brain Epigenetic Concordance (BECon) tool^15^. BECon utilizes paired samples (N=16) to quantify the correlation between blood and brain across three Brodmann brain areas. The output provides information on inter-individual variability, blood-brain DNAm correlation, and estimations of the effect of cell composition on blood and brain DNAm. To identify probes with good blood-brain concordance, we used the following criteria: percentile of mean positive correlation across all three Brodmann regions is greater than 50%, variability in DNAm levels is present across blood and all brain regions using a threshold of 0.05.

#### Methylation Quantitative Trait Loci (mQTL) Mapping

To quantify the potential genetic influence on DNAm levels at significant CpG sites, we identified mQTLs using the GoDMC API ^16^. GoDMC is a database comprising of mQTLs from over 32,000 participants from 36 cohorts using the 450K BeadChip. We filtered the results by a stringent p-value threshold of 1e-14, as the authors recommended.

## **Socioeconomic status (SES) composite variable**

The SES composite variable was calculated as follows:

**INPUTS**

STEP 1: RESPONSE CODING

- 1. Maternal employment
     1. Employment = 1 vs Unemployment = 0
  2. Maternal education
     1. Primary education = 0
     2. Some secondary = 1
     3. Completed secondary = 2
     4. Any tertiary = 3
  3. Household income
     1. <R1000/m = 0
     2. R1000 – 5000/m = 1
     3. >R5000/m =2
  4. Household assets and financial activities
     1. Sum over 10 items (each checked item counts as 1):
        1. Electricity
        2. Tap or running water
        3. Domestic worker
        4. A flush toilet inside
        5. A built-in kitchen sink
        6. An electric stove or hotplate
        7. A working telephone (this includes a cell phone)
        8. At least one motor car or truck
        9. A motorcycle or scooter
        10. A bicycle
     2. Sum over 3 items (each checked item counts as 1)
        1. Shop at supermarkets
        2. Use any financial services (such as bank account, ATM card or credit card)
        3. Have an account at a retail store (e.g. Pep, Jet, etc)

STEP 2: STANDARDIZATION OF INPUTS

1. Standardize the sum of household asset/financial activities (variable name: ***“stdassetsum”***)
2. Standardize income (variable name: ***“stdincome”***)
3. Standardize education (variable name: ***“stdeducation”***)

**OUTPUTS**

STEP 1: DERIVING A *CONTINUOUS* SOCIOECONOMIC COMPOSITE

1. Continuous socioeconomic status composite (variable name: *“****sessumscore****”*)

**Sessumscore** = **stdassetsum** + **stdeducation** + **stdincome** + **(employment*0.5)**

STEP 2: DERIVING A *CATEGORICAL* SOCIOECONOMIC COMPOSITE

1. Categorical socioeconomic status composite (variable name: *“****sesquartile****”*)

**Sesquartile** is derived from **sessumscore** composite split into **quartiles**:

- - - 1. Lowest SES
      2. Low-mod SES
      3. Mod-high SES
      4. High SES

**
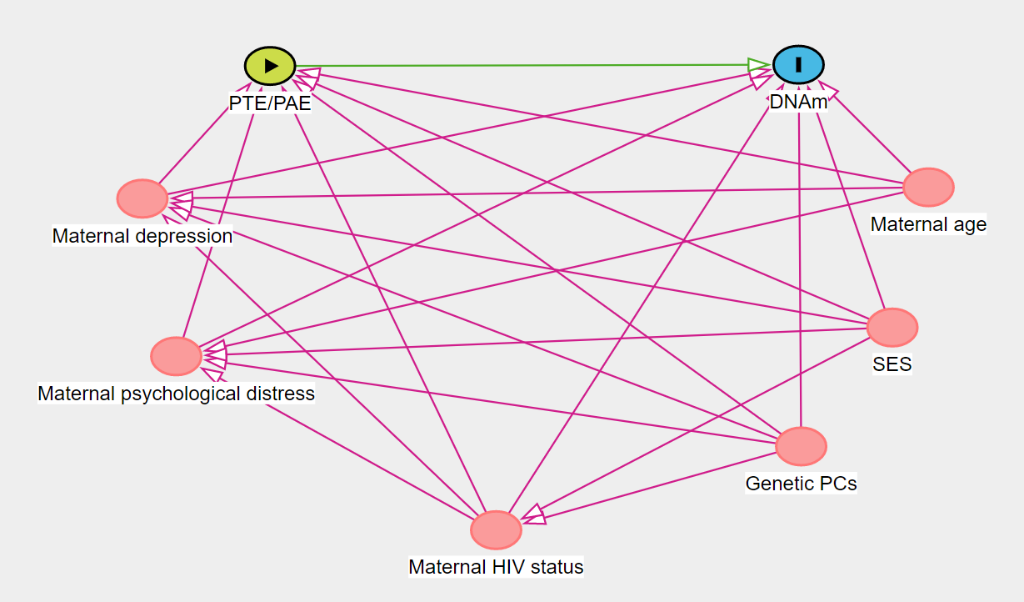
**

**A**


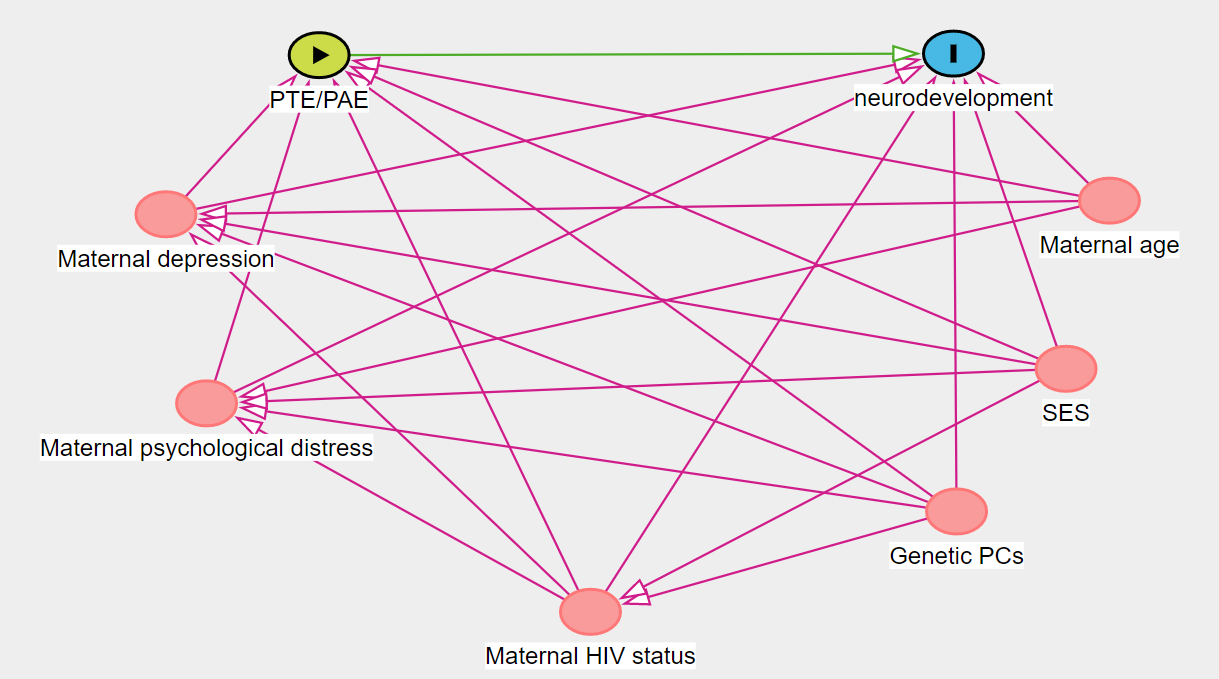


**B**


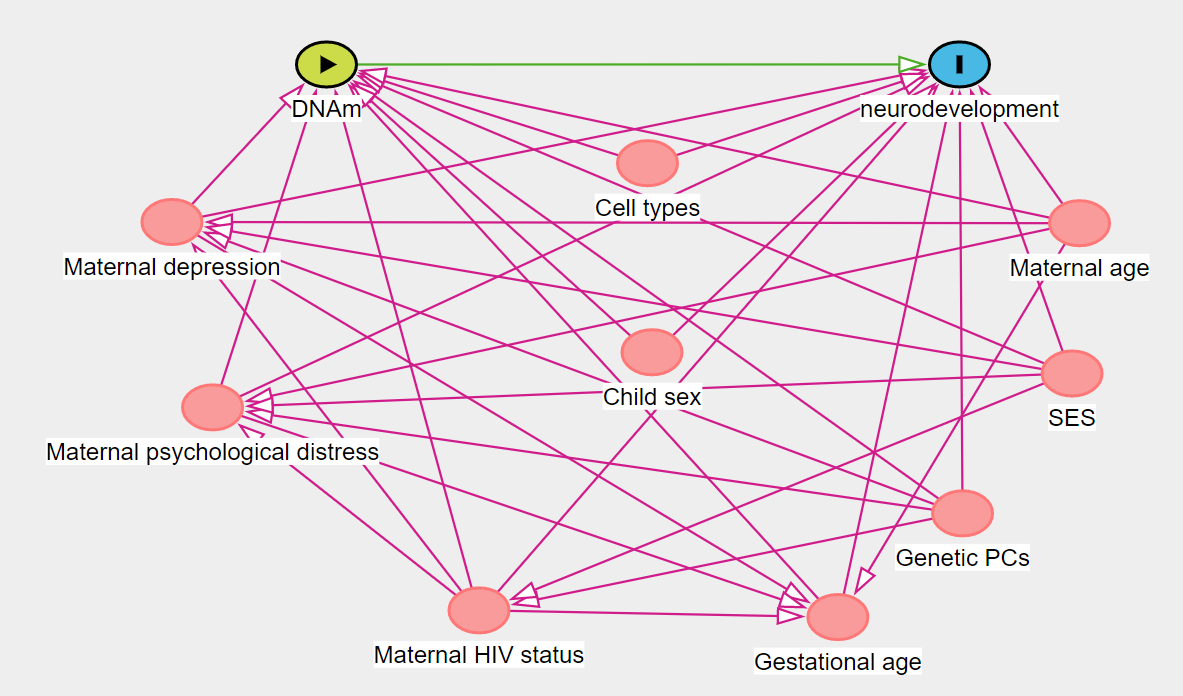


**C**

**Figure S1:** Directed acyclic graphs (DAGs) used for confounding assessment. **S1A)** Exposure-mediator DAG. **S1B)** Exposure-outcome DAG. **S1C)** Mediator-outcome DAG


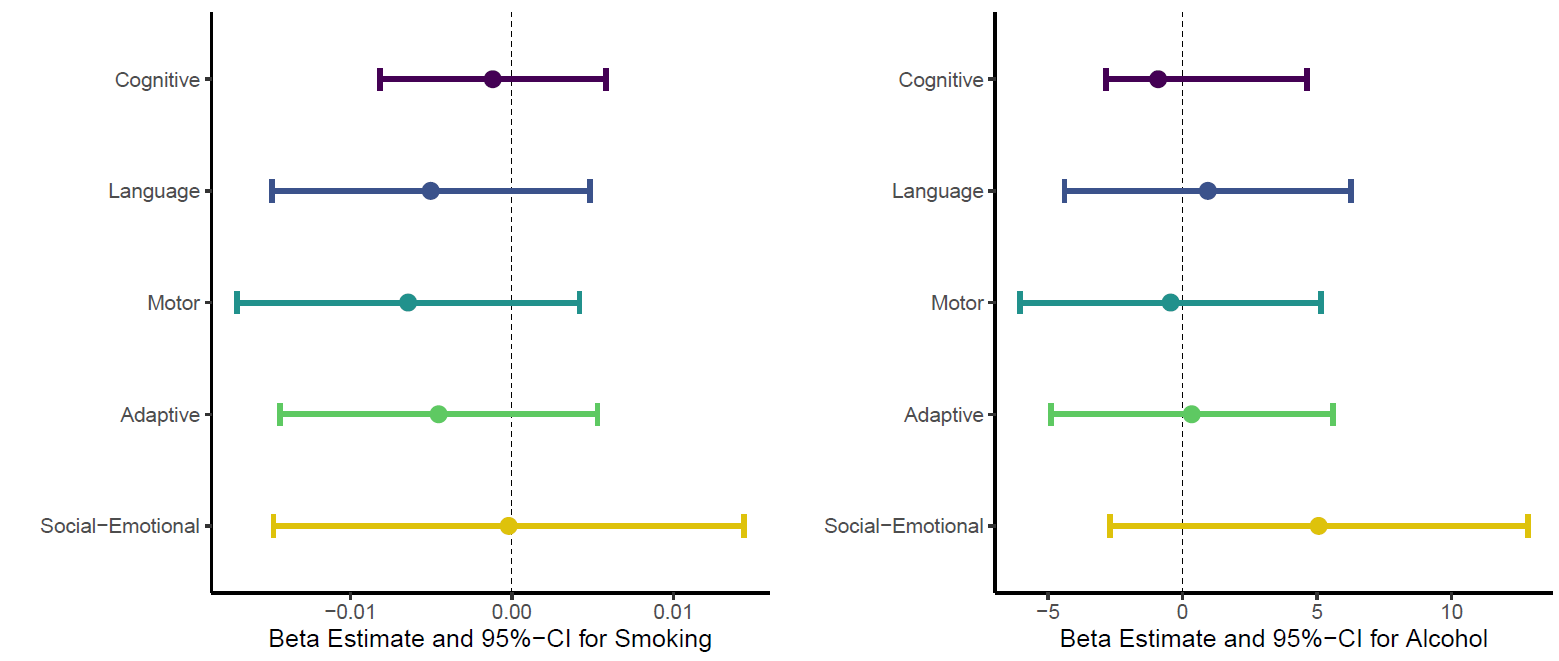


**B**

**A**

**Figure S2:** Association of PTE and PAE on neurodevelopment domains at 24 months. Models were adjusted for parental SES, maternal depression, maternal psychological distress, gestational age, maternal age, maternal HIV status, cell-type proportions, and the first 5 genetic principal components. **S2A)** Association of PTE on neurodevelopment domains, additionally adjusted for PAE. **S2B)** Association of PAE on neurodevelopment domains, additionally adjusted for PTE.

**References**

1 Morin AM, Gatev E, McEwen LM, Macisaac JL, Lin DTS, Koen N *et al.* Maternal blood contamination of collected cord blood can be identified using DNA methylation at three CpGs. *Clin Epigenetics* 2017; **9**: 1–9.

2 R Core Team. R: A language and environment for statistical computing. 2018.

3 Aryee MJ, Jaffe AE, Corrada-Bravo H, Ladd-Acosta C, Feinberg AP, Hansen KD *et al.* Minfi: A flexible and comprehensive Bioconductor package for the analysis of Infinium DNA methylation microarrays. *Bioinformatics* 2014; **30**: 1363–1369.

4 Fortin J-P, Labbe A, Lemire M, Zanke BW, Hudson TJ, Fertig EJ *et al.* Functional normalization of 450k methylation array data improves replication in large cancer studies. *Genome Biol* 2014; **15**: 503.

5 Leek JT, Johnson WE, Parker HS, Jaffe AE, Storey JD. The sva package for removing batch effects and other unwanted variation in high-throughput experiments. *Bioinformatics* 2012; **28**: 882–883.

6 Gervin K, Salas LA, Bakulski KM, Van Zelm MC, Koestler DC, Wiencke JK *et al.* Systematic evaluation and validation of reference and library selection methods for deconvolution of cord blood DNA methylation data. *Clin Epigenetics* 2019; **11**: 1–15.

7 Koestler DC, Jones MJ, Usset J, Christensen BC, Butler RA, Kobor MS *et al.* Improving cell mixture deconvolution by identifying optimal DNA methylation libraries (IDOL). *BMC Bioinformatics* 2016; **17**: 1–21.

8 Du P, Kibbe WA, Lin SM. lumi: A pipeline for processing Illumina microarray. *Bioinformatics* 2008; **24**: 1547–1548.

9 Hannum G, Guinney J, Zhao L, Zhang L, Hughes G, Sadda S *et al.* Genome-wide methylation profiles reveal quantitative views of human aging rates. *Mol Cell* 2013; **49**: 359–367.

10 Efron B, Brit Turnbull B, Narasimhan B. locfdr: Computes Local False Discovery Rates. R package version 1.1-8. 2015.

11 Pidsley R, Y Wong CC, Volta M, Lunnon K, Mill J, Schalkwyk LC. A data-driven approach to preprocessing Illumina 450K methylation array data. *BMC Genomics* 2013; **14**. doi:10.1186/1471-2164-14-293.

12 Pidsley R, Zotenko E, Peters TJ, Lawrence MG, Risbridger GP, Molloy P *et al.* Critical evaluation of the Illumina MethylationEPIC BeadChip microarray for whole-genome DNA methylation profiling. *Genome Biol* 2016; **17**: 1–17.

13 Price ME, Cotton AM, Lam LL, Farré P, Emberly E, Brown CJ *et al.* Additional annotation enhances potential for biologically-relevant analysis of the Illumina Infinium HumanMethylation450 BeadChip array. *Epigenetics Chromatin* 2013; **6**: 1–15.

14 Phipson B, Maksimovic J, Oshlack A. MissMethyl: An R package for analyzing data from Illumina’s HumanMethylation450 platform. *Bioinformatics* 2016; **32**: 286–288.

15 Edgar RD, Jones MJ, Meaney MJ, Turecki G, Kobor MS. BECon : a tool for interpreting DNA methylation fi ndings from blood in the context of brain. 2017; **7**: e1187-10.

16 Min JL, Hemani G, Hannon E, Dekkers KF, Castillo-Fernandez J, Luijk R *et al.* Genomic and phenotypic insights from an atlas of genetic effects on DNA methylation. *Nat Genet* 2021; **53**: 1311–1321.
